# Beneficial Effect of Higher Dietary Fiber Intake on Plasma HDL-C and TC/HDL-C Ratio among Chinese Rural-to-Urban Migrant Workers

**DOI:** 10.3390/ijerph120504726

**Published:** 2015-04-29

**Authors:** Quan Zhou, Jiang Wu, Jie Tang, Jia-Ji Wang, Chu-Hong Lu, Pei-Xi Wang

**Affiliations:** 1 Faculty of Preventive Medicine, School of Public Health, Guangzhou Medical University, Guangzhou, 195# West Dongfeng Road, Guangzhou 510182, China; E-Mails: joan_zq@126.com (Q.Z.); gytanjie@163.com (J.T.); wjia@163.com (J.W.); chuhon688@163.com (C.L.); 2 Shenzhen Baoan City Center Hospital, 6 Xiyuan Road, Baoan District, Shenzhen 518102, China; E-Mail: WuJiang731@163.com

**Keywords:** dietary fiber, high-density lipoprotein cholesterol, total cholesterol to high-density lipoprotein cholesterol ratio

## Abstract

Research has shown that high-dose supplemental dietary fiber intake has beneficial effects on cardiovascular risk factors. To clarify such a relationship, we examined the association between daily dietary fiber intake and plasma lipids using a cross-sectional design including 1034 (M 502, F 532) rural-to-urban workers in China. We found a dose-response relationship between increased dietary fiber intakes and increase of HDL cholesterol in male workers. There was also a dose-response relationship between increased dietary fiber intake and decreased total cholesterol to HDL cholesterol (TC/HDL-C) ratio in both male and female workers, after adjusting for potential confounders (*p* for trend, all *p* < 0.05). When the average dietary fiber intake increased from less than 18 g/day to over 30 g/day, the average HDL cholesterol level increased by 10.1%, and the TC/HDL-C ratio decreased by 14.4% for males (*p* = 0.020) and by 11.1% for females (*p* = 0.048). In conclusion, higher daily dietary fiber consumption is associated with beneficial effect on cholesterol for rural-to-urban workers in China, suggesting its potential beneficial effect on decreasing the risk of cardiovascular diseases.

## 1. Introduction

Cardiovascular disease (CVD) remains the leading cause of mortality worldwide and caused about 17.5 million death in 2012 [1]. The World Health Organization (WHO) estimates that 75% of cardiovascular mortality could be decreased with appropriate lifestyle changes; and this is the biggest challenge presented by the various guidelines on CVD prevention [2].

One strategy that has been attempted for this purpose is increasing the dietary fiber (DF) intake. As the residue of plant food resistant to hydrolysis by human alimentary enzymes, DF is a non-digestible carbohydrate and includes a heterogeneous mixture of polysaccharides and lignin [3], offering potential health benefits to humans [4]. Since the first finding that dietary fiber has some beneficial effects on ischaemic heart disease at the 1970s [5], lots of observational and experimental studies have explored the relationship between dietary fiber or fiber-rich foods and cardiovascular diseases or cardiovascular diseases risk factors, like obesity, hypertension and dyslipidemia [6,7,8].

Total cholesterol to high-density lipoprotein cholesterol (TC/HDL-C) ratio is a high discriminatory power index for coronary heart disease. High TC/HDL-C ratio has been intensively used as a predictor of CVD. Previous studies have reported a beneficial effect of DF intake on TC/HDL-C ratio, but the average DF intake in those studies was about 48 g/day [9], much higher than the average Chinese DF intake of 18 g/day [10].

High-density lipoprotein (HDL) plays a role in reverse cholesterol transport, reveals that HDL may suppress cholesterol accumulation in the peripheral tissues. Both epidemiological and basic science studies have shown an inverse association between the concentration of plasma HDL cholesterol (HDL-C) and the risk of developing atherosclerotic CVD [11]. According to results of meta-analyses, whether DF intake has beneficial effects on HDL-C could not be determined [12]. Yet the result of clinical trials performed in Asian populations suggested that DF intake may help people maintain or increase their plasma HDL-C values [13].

The differences of DF intake amount may partially explain the different results. The “Dietary Guidelines for Chinese Residents” recommends a DF intake of 30 g/day [14], higher than that of WHO recommendation (25 g/day) [15]. To date, there is little research evaluating whether this amount of DF intake has any beneficial effects on blood lipid profiles in a Chinese population. Hence, this study was undertaken to evaluate the association of DF intake with plasma lipids in rural-to-urban migrant workers.

## 2. Experimental Section

### 2.1. Study Subjects

This is a sub-study of the 2013 rural-to-urban migrant workers study performed in the Shenzhen-Dongguan economic zone of China, which was approved by the Research Ethics Board of Guangzhou Medical University (Guangzhou, China). The detailed methodology of this study is briefly described below. Three factories (a textile manufacturer, a furniture assembler and a camera assembler) were approached and they agreed to the implementation of the study protocol. Migrant workers between 18 and 59 years of age in the three factories were sampled by a cluster randomization method. One third of workshops in the three factories were randomly sampled. All subjects working in the sampled workshops were recruited (*n* = 2315 rural-to-urban migrant workers). Among them, all subjects (*n* = 1228) from the Dongguan camera assembler were invited to take a nutritional survey. Of these, 1127 participated in the present study (response rate, 91.8%). Subjects who were taking lipids or blood glucose-lowering drugs and those with a history of cardiovascular diseases (*n* = 31) were excluded. Finally, a total of 1096 participants were included for the present study.

### 2.2. Data Collection

#### 2.2.1. Questionnaire Interview

Information on sociodemographic data, health history, general risk factors of CVD, physical activities, current dietary intakes, and female reproductive history were collected by trained interviewers using a face-to-face interviewing of structured questionnaire. Questionnaire interview was conducted prior to laboratory assay.

#### 2.2.2. Dietary Assessment

Participants completed a three-day diet record, in which they noted all foods and drinks during two weekdays and one weekend day [16]. Food models in the reference portion sizes and standard tablewares were provided as visual aids to help participants weigh the amount of foods and drinks they consumed. If weighing was not possible, participants were instructed to estimate the amount of foods and drinks they consumed by using standard household measures (e.g., spoon, glass, cup, *etc*.). Intake of dietary fiber and other nutrients was calculated based on the Chinese Food Composition Table (data of soluble and insoluble fiber are not discriminated) [17]. Energy-adjusted DF intake (g/1000 kcal/day) was calculated using the energy density method [18]. In the present study, individuals who reported unrealistic energy intake (<500 or >5000 kcal/day, *n* = 62) were excluded from the final data analyses.

#### 2.2.3. Anthropometric and Blood Pressure Measurement

The height and weight of participants were measured to the nearest 0.5 cm and 0.1 kg respectively. Body mass index (BMI) was then calculated as weight (kg)/height (m)^2^. Waist circumference (WC) was measured half way between the lowest rib margin and the iliac crest, and hip circumference was measured at the level of the greater trochanters. Measurements of WC and hip circumference were performed twice and the average values were used for analyses. Waist to hip ratio (WHR) was then calculated. Two consecutive measurements of blood pressure were taken from the right arm after subjects had been sitting for at least 10 min. Systolic blood pressure (SBP) and diastolic blood pressures (DBP) were recorded to the nearest 2 mm Hg. If the two systolic or diastolic blood pressures recorded differed ≥4 mmHg, a third measurement was made. The average value of the two blood pressures was used for analyses. SBP and DBP were defined as the point of the appearance (Korotkoff I) and disappearance (Korotkoff V) of Korotkoff sounds, respectively.

#### 2.2.4. Laboratory Assay

The 12-h fasting venous blood was collected with vacuum tubes containing EDTA for lipids analysis. Plasma was separated after centrifugation at 1500 × *g* for 15 min at 4  C within 2 h and stored at −80  C till tests. All samples were analyzed in a single batch within 5 days to minimize laboratory variability. Plasma TC, TG, HDL-C and LDL-C were measured with colorimetric methods using commercial kits (Biosino Biotechnology Company Ltd., Beijing, China) by an automated analyzer (A25 Biosystem, Barcelona, Spain). The coefficients of variation for lipid measurements were 2.3% (at 4.50 mmol/L TC), 5.8% (at 1.77 mmol/L TG), 4.3% (at 1.28 mmol/L HDL-C), and 3.1% (at 3.29 mmol/L LDL-C). TC/HDL-C was calculated as TC (mmol/L)/HDL-C (mmol/L). Laboratory assay was conducted by two staffs which were not involved in the questionnaire interview and did not know the exposure status of the subjects.

### 2.3. Statistical Analysis

Results were presented as mean (S.D.). Mean dietary intakes were corrected for within-person variation by means of the multiple source method (MSM) [19]. Based on the average DF intake of Chinese (18 g/day) and the recommended daily dietary fiber intake in China (30 g/day) participants were categorized into either >30 g/day (high DF intake group, HI group), between 18 g/day and 30 g/day (median DF intake group, MI group), or <18 g/day (low DF intake group, LI group). Comparisons of characteristics among the three groups were performed using either one-way ANOVA or χ^2^ test. The statistical significances of pairwise comparisons were corrected by Bonferroni test. Mean differences in plasma lipids and blood glucose among the three dietary fiber groups were compared using analysis of covariance (ANCOVA), after adjusting for age, BMI, waist circumference, smoking, total dietary energy, dietary fat and protein, and energy from saturated fat. Assumptions of tests were examined and confirmed prior to analyses. Two-way analysis of covariance was used to examine the interaction between sex and the three levels of dietary fiber intake on plasma lipids. All analyses were conducted with SPSS for Windows (version 13.0, SPSS, Inc., Chicago, IL, USA). A two-sided *p-*value of less than 0.05 was considered as statistically significant.

## 3. Results and Discussion

### 3.1. Results

The characteristics of the 1034 participants are shown in Table 1. The mean (S.D.) ages of female and male participants were 36.7 (8.1) and 36.4 (7.5) years, respectively. There was no significant difference in BMI between male and female workers, significant differences were found in waist circumstance, hip circumstance and waist to hip ratio.

**Table 1:** Characteristics (mean ± S.D.) of the 1034 rural-to-urban migrant workers.

	Men (*n* = 134)	Women (*n* = 272)	*p-*Value
Age (year)	36.7 ± 8.1	36.4 ± 7.5	0.628
Weight (kg)	60.8 ± 9.1	55.8 ± 8.9	<0.001
Height (cm)	161.2 ± 7.0	152.9 ± 6.2	<0.001
Body mass index (kg/m^2^)	24.1 ± 3.2	23.8 ± 3.3	0.235
BMI category (%)			0.043
Underweight	0.5	2.7	
Normal weight	53.9	57.1	
Overweight	34.7	29.0	
Obesity	10.4	11.3	
Waist circumference (cm)	77.2 ± 8.1	75.5 ± 8.8	0.006
Hip circumference (cm)	89.0 ± 5.7	90.1 ± 6.4	0.016
Waist to hip ratio	0.9 ± 0.06	0.8 ± 0.1	<0.001
Systolic blood pressure (mmHg)	119.8 ± 15.7	115.3 ± 13.9	<0.001
Diastolic blood pressure (mmHg)	73.6 ± 13.1	70.6 ± 9.8	<0.001
Smoking			<0.001
Non-smoker (%)	40.5	98.9	
Ex-smoker (%)	6.3	0.0	
Current-smoker (%)	53.2	1.1	
Diet intake			
Energy (kcal/day)	1874.8 ± 694.9	1657.0 ± 638.8	<0.001
Protein (g/day)	62.5 ± 34.4	54.0 ± 32.2	0.001
Total fat (g/day)	70.3 ± 36.1	64.2 ± 31.6	0.120
Saturated fatty acids (%kcal)	14.6 ± 12.8	15.4 ± 11.7	0.344
Polyunsaturated fatty acids (%kcal)	9.7 ± 6.7	10.6 ± 7.4	0.079
Energy adjusted dietary fiber (g/1000 kcal)	9.5 ± 6.7	10.0 ± 6.2	0.298
Total dietary fiber (g/day)	15.5 ± 8.0	14.5 ± 7.0	0.061
Total dietary fiber category			0.313
~18 g/day	73.9	78.6	
~30 g/day	20.5	17.1	
≥30 g/day	5.6	4.3	
Serum biomarkers			
Total cholesterol (mmonl/L)	4.6 ± 0.9	4.4 ± 0.8	0.006
HDL cholesterol (mmol/L)	1.4 ± 0.3	1.5 ± 0.3	<0.001
LDL cholesterol (mmol/L)	2.2 ± 0.5	2.1 ± 0.5	0.001
Triglycerides (mmol/L)	1.5 ± 0.9	1.0 ± 0.8	<0.001
TC to HDL-C ratio	3.4 ± 0.9	3.1 ± 0.7	<0.001
Blood Glucose (mmol/L)	5.0 ± 0.9	5.1 ± 0.9	0.427

**Figure 1:**
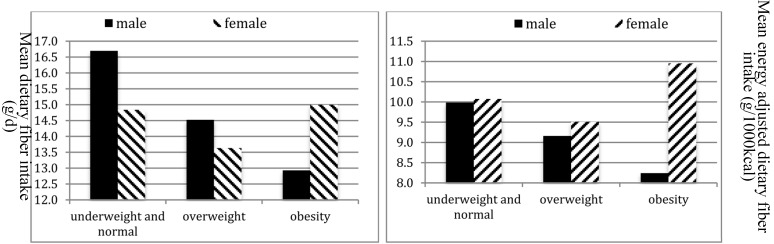
Mean (**a**) total and (**b**) energy-adjusted dietary fiber intakes by BMI category stratified by gender of rural-to-urban participants.

The mean (S.D.) dietary fiber intakes in female and male workers were 15.5 (8.0) and 14.5 (7.0) g/day, and the mean energy-adjusted DF intakes were 9.5 (6.7) and 10.0 (6.2), respectively. The majority of subjects consumed less than the average amount of DF (76.2%), including 73.9% males and 78.6% females. Only 5% subjects consumed the recommended daily intake level including 5.6% males and 4.3% females. Males had significantly higher intake of energy (*p* < 0.001) and protein (*p* = 0.001) than females. Furthermore, over-weight (OW) and obese males consumed less total DF compared with under- weight (UW) and normal-weight (NW) peers (Figure 1). No significant differences were observed in total and energy-adjusted DF intakes among females of different BMI categories.

Univariate analysis showed a dose-response relationship between increased HDL-C and increased DF intake in male workers (*p* for trend 0.004). For male workers the mean HDL-C was increased by 11.3% in HI group comparing with that in LI group. And a negative correlation between DF intake and TC/HDL-C ratios in male (*p* for trend 0.004) and female (*p* for trend 0.022) workers were found, the mean TC/HDL-C ratio was decreased by 17.5% for male workers and 12.7% for females in HI group comparing with LI group. No significant differences of TC, LDL-C, BP, triglycerides and blood glucose among the three DF intake groups were observed in both male and female workers (all *p* > 0.05). Higher DF intake was also associated with higher intakes of total protein and total fat, but there was significantly lower daily intake of total carbohydrates in this sample of individuals (Table 2).

**Table 2:** Comparison of means of parameter by three DF intake Groups.

	Low			Mid			High		% Difference (high *vs*. low)	*p* for one-way ANOVA	*p* for linear trend
	Mean	S.D.		Mean	S.D.		Mean	S.D.			
Men (*n* = 502)										
Systolic BP (mmHg)	120.2	16.2		119.9	15.4		120.8	14.9	0.5	0.971	0.873
Diastolic BP (mmHg)	73.6	13.2		74.7	12.3		76.7	11.8	4.0	>0.511	0.306
Total cholesterol (mmol/L)	4.6	0.9		4.6	1.0		4.4	0.9	−4.1	>0.599	0.379
HDL cholesterol (mmol/L)	1.3	0.3		1.4	0.3		1.5 *	0.3	11.3	>0.014	0.006
LDL cholesterol (mmol/L)	2.2	0.5		2.2	0.5		2.0	0.5	−8.8	>0.301	0.135
TC/HDL-C	3.5	0.9		3.4	0.8		3.0 *	0.5	−17.5	0.011	0.004
Triglycerides (mmol/L)	1.5	0.8		1.6	1.0		1.6	0.8	7.4	0.841	0.613
Blood Glucose (mmol/L)	5.0	0.9		5.0	0.7		4.8	0.6	−3.7	0.642	0.347
Age (year)	36.5	8.3		37.5	7.7		36.2	8.9	−1.1	0.630	0.841
Body mass index (kg/m^2^)	24.5	3.3		23.2 *	2.5		21.8 *	2.8	−12.2	<0.001	<0.001
Waist circumference (cm)	77.7	8.9		77.7	8.9		75.7	6.9	−2.6	0.613	0.323
Waist-to-hip ratio	0.9	0.1		0.9	0.1		0.9	0.1	−1.2	0.970	0.807
Total dietary energy (kcal/day)	1875	725		1887	701		2093	861	10.4	0.423	0.190
Total dietary protein (g/day)	54.9	27.1		80.2 *	45.4		100.2 *	34.5	45.2	<0.001	<0.001
Total dietary carbohydrate (g/day)	427.9	338.5		259.7 *	125.2		206.1 *	97.6	−107.6	<0.001	<0.001
Total dietary fat (g/day)	64.9	48.8		86.2 *	73.9		90.7	61.7	28.4	0.003	0.041
Dietary saturated fat (% of energy)	13.8	9.8		17.4	20.6		16.2	9.5	15.0	0.081	0.400
Dietary polyunsaturated fat (% of energy)	9.0	4.6		12.2 *	9.6		11.1	5.9	18.4	<0.001	0.140
Women (n = 532)										
Systolic BP (mmHg)	115.7	14.6		117.1	16.1		113.0	10.4	−2.4	0.594	0.473
Diastolic BP (mmHg)	70.8	10.5		71.8	9.5		71.3	9.0	0.6	0.793	0.867
Total cholesterol (mmol/L)	4.5	0.8		4.4	0.7		4.5	0.9	1.3	0.492	0.758
HDL cholesterol (mmol/L)	1.3	0.3		1.5	0.3		1.5	0.2	11.5	0.089	0.031
LDL cholesterol (mmol/L)	2.1	0.5		1.9	0.5		2.3	0.6	7.1	0.025	0.201
TC/HDL-C	3.6	0.7		3.0	0.5		3.2 *	0.9	−12.7	0.021	0.022
Triglycerides (mmol/L)	1.0	0.8		1.2	0.9		1.1	0.8	12.3	0.369	0.508
Blood Glucose (mmol/L)	5.0	0.8		5.0	0.6		5.1	0.6	0.6	0.892	0.887
Age (year)	36.5	7.4		36.3	7.6		35.4	8.4	−3.1	0.831	0.554
Body mass index (kg/m2)	23.8	3.2		23.7	3.9		23.6	2.8	−1.0	0.918	0.772
Waist circumference (cm)	76.0	9.4		73.4	9.5		76.7	9.0	0.9	0.119	0.765
Waist-to-hip ratio	0.8	0.1		0.8	0.1		0.8	0.1	0.0	0.204	0.888
Total dietary energy (kcal/day)	1646	656		1687	680		1708	848	3.6	0.848	0.715
Total dietary protein (g/day)	49.3	28.0		67.4 *	41.1		83.9 *	34.5	41.2	<0.001	<0.001
Total dietary carbohydrate (g/day)	270.7	119.2		223.5 *	81.3		178.3*	79.6	−51.8	<0.001	<0.001
Total dietary fat (g/day)	62.0	48.7		64.3	56.7		62.0	48.7	0.0	0.105	0.034
Dietary saturated fat (% of energy)	14.8	9.9		16.2	16.3		19.9	15.6	25.5	0.181	0.088
Dietary polyunsaturated fat (% of energy)	9.8	5.0		12.1	11.3		18.2 *	15.6	46.1	<0.001	<0.001

* *p* < 0.05compared with the low intake group (Bonferroni).

Analysis of covariance was conducted to examine whether there was an independent association between daily DF intake and blood lipids and glucose after adjusting for potential confounders, such as age, BMI, waist circumference, smoking, total dietary energy, intakes of protein, fat, saturated fatty acids and polyunsaturated fatty acids. Similar associations were observed (Table 3). The mean value of HDL-C was 10.1% higher in male workers (*p* = 0.02), and the TC/HDL-C ratio was 14.4% (*p* = 0.028) and 11.1% (*p* = 0.048) lower in male and female workers, respectively, when comparing the HI group with the LI group of DF intake (Table 3).

### 3.2. Discussion

In this cross-sectional study, we found that increased DF intake was significantly associated with an increase plasma HDL-C level in male workers after adjusting for confounding factors (e.g., age, BMI, waist circumference, smoking, total dietary energy, intakes of protein, fat, saturated fatty acids and polyunsaturated fatty acids), as well as for a decreased TC/HDL-C ratio in male and female workers. HDL-C was increased by 10.1% for male and TC/HDL-C ratio was decreased by 14.4% and 11.1% for males and females respectively, when daily DF intake was higher than 30 g/day. 

HDL is thought to function as a sterol transporter that facilitates the movement of sterols from peripheral cells to the liver. High HDL-C level is associated with a reduced risk of coronary heart disease [20,21]. Recently, Schaffer *et al*. [22] reported that the relative risk of coronary artery disease was 1.48 (95% CI, 1.37–1.6) in subjects with HDL-C < 32 mg/dl as compared with the highest quintile of HDL-C (>47 mg/dl) after adjusting for potential confounders in a cohort study. Our findings also implied that higher daily DF intake might be associated with a lower risk of CVD in Chinese rural-to-urban male workers.

**Table 3:** Covariable-adjusted means of parameters by three DF intake Groups.

	Low		Median		High		%Difference (high *vs*. low)	*p* for ANCOVA	*p* for linear trend
	Mean	S.E.M	Mean	S.E.M	Mean	S.E.M			
Men (*n* = 502) ^a^									
Total cholesterol (mmol/L)	4.6	0.04	4.5	0.10	4.5	0.19	−3.4	0.585	0.472
HDL cholesterol (mmol/L)	1.3	0.02	1.4	0.03	1.5 *	0.06	10.1	0.020	0.007
LDL cholesterol (mmol/L)	2.2	0.03	2.2	0.06	2.0	0.11	−8.9	0.316	0.135
TC/HDL-C	3.5	0.05	3.3	0.09	3.1 *	0.17	−14.4	0.028	0.014
Triglycerides (mmol/L)	1.5	0.06	1.6	0.14	1.7	0.27	9.0	0.361	0.184
Blood Glucose (mmol/L)	5.0	0.04	5.0	0.10	4.8	0.19	−3.7	0.939	0.940
Systolic BP (mmHg)	120.3	0.76	119.8	1.79	123.6	3.45	2.7	0.603	0.339
Diastolic BP (mmHg)	73.7	0.62	74.4	1.46	78.1	2.82	5.7	0.298	0.127
Women (*n* = 532) ^a^									
Total cholesterol (mmol/L)	4.4	0.04	4.4	0.10	4.5	0.19	1.1	0.831	0.689
HDL cholesterol (mmol/L)	1.4	0.01	1.4	0.03	1.5	0.07	6.9	0.112	0.037
LDL cholesterol (mmol/L)	2.1	0.02	1.94	0.06	2.2	0.12	6.7	0.034	0.233
TC/HDL-C	3.5	0.04	3.1	0.08	3.1 *	0.17	−11.1	0.048	0.024
Triglycerides (mmol/L)	1.0	0.03	1.0	0.09	1.0	0.18	−1.0	0.786	0.519
Blood Glucose (mmol/L)	5.0	0.04	5.1	0.10	5.0	0.20	0.3	0.803	0.508
Systolic BP (mmHg)	115.4	0.68	117.6	1.85	113.6	3.51	−1.5	0.450	0.624
Diastolic BP (mmHg)	70.8	0.49	72.0	1.33	71.1	2.52	0.5	0.688	0.900

^a^ Adjusted for age, BMI, waist circumference, smoking status and energy, protein, saturated fat, polyunsaturated fat, and carbohydrate intake; * *p* < 0.05 compared with the low intake group (Bonferroni).

To date, studies examining the association between DF intake and risk of CVD have yielded inconsistent results. A sub-study of the Japan Collaborative Cohort Study including 58,730 adults aged 40–79 y showed that covariates-adjusted CVD risk was 0.79 (95% CI: 0.61–0.98) in subjects consuming a high DF of >12.6 g/d as compared with those consuming a low intake of <7.8 g/day [23]. Yet a prospective study in the EU reported no statistically significant difference in CVD risk among quintile groups of DF intake (from 14.1 g/day to 38.3 g/day) after a 14.3-year follow-up of 31,036 females [24]. Our findings seem to support a favorable association between DF intake and a lower CVD risk. The reason for the differences among these observational studies was unclear. The different sources of DF in ethnic foods may contribute in part to the contradictory results. Hopping *et al*. [25] reported that the protective effects of dietary fiber on the development of diabetes differed by ethnic group according to consumed foods, which may be a plausible theory applying to cardiovascular diseases as well. 

It has been established that high plasma TC, LDL-C, TG, TC/HDL-C and low HDL-C increase risk of CVD. Randomized controlled trials in Asia had found that high-dose DF supplemental generally had a favorable effect on serum lipid profiles. In the present study, TC, LDL-C and TG were not associated with dietary fiber intake. We observed a significant difference of HDL-C among the three groups of daily DF intake in rural-to-urban male workers. We also observed significant differences of TC/HDL-C ratios in both male and female workers. 

Studies evaluating the relationship between dietary fiber and HDL-C have given inconsistent results. Studies performed in Asian populations supported that dietary fiber was beneficially associated with HDL-C, while randomized controlled trials in patients with hypertension [26,27] suggested that increased intake of soluble dietary fiber was not associated with increased HDL-C. Another trial using refined konjac meal as source of dietary fiber yielded a similar result [28]. A meta-analysis [12] showed that the use of barley did not significantly alter HDL-C (*p* = 0.07) in healthy and hypercholesterolemic individuals. A recent trial performed in China investigated the impact of oat consumption on cholesterol levels in Chinese adults with mild to moderate hypercholesterolemia, the result of which indicated that the HDL-C level was unchanged in oat group which is rich in dietary fiber after 6 weeks intervention [13]. In the present study, we found that a dietary fiber intake of over 30 g/day might be beneficial to males for improving their plasma HDL-C. 

TC/HDL-C ratio is more sensitive in reflecting the morbidity and severity of CVD than individual lipid levels. A randomized trial performed by Reid *et al*. [29] reported that subjects who consumed one-third cup per day of psyllium-containing cereal and were advised to increase soluble fiber intake to over 10 g/day for 26 weeks gained a 4.6% TC/HDL-C ratio reduction. Similar results were found in another clinical trial, in which 4 weeks of hydroxypropylmethyl cellulose consumption (5 g/day) resulted in significant reductions of TC/HDL-C ratio along with TC, non-HDL-C and LDL-C [30]. Maki *et al*. [31] provided 3 g/day of β-glucan from whole-grain ready-to-eat oat cereal to overweight and obese adults and observed reductions in TC and LDL-C as early as week 4. Consistent with the meta-analysis by Kelly *et al*. [32], which indicated that oat consumption is associated with lower TC (*p* = 0.005) and LDL-C concentrations (*p* = 0.008), Ballesteros *et al*. [9] also reported a beneficial effect of high DF intake (48 g/day) on plasma TC, LDL-C and TG in adult men. In the present study, TC/HDL-C ratio was negatively associated with total dietary fiber intake. However, we did not observe significant association between dietary fiber intake and TC, LDL-C and TG, which is possibly due to limited sample size or a large random error in fiber intake measurement.

Interestingly in the current study we detected that OW and obese males consumed less total DF compared with UW and NW males. Although DF has been used in the prevention or treatment of obese in children and adults [33], conflicted results about the association between dietary fiber intake and body weight were reported. Previous findings [34] suggested that DF intake during puberty might not affect the concurrent development of body fat percentage or BMI. However, Pal *et al*. [35] reported that after supplement with fiber for 12 weeks, subjects had a significantly reduced body weight and BMI along with great improvements in serum biomarkers of CVD. Recently, Lin *et al*. [36] reported that OW and obese females consumed less total and energy-adjusted DF compared with UW and NW peers. Another study [37] suggested that total DF intake was significantly lower in Swiss OW boys, but not girls. In present study the tendency of total DF intake throughout the weight groups shows that obese female workers had the highest consumption of DF. However, differential misreporting (e.g., more underreporting among the obese) could bias those results.

Some limitations of this study need to be considered. The major limitation of this study is the cross-sectional study design, in which causal relationship is hard to be identified due to no clear time–sequence relationship between the relevant exposures and outcomes. Moreover, inverse causal relationships are often observed if the exposures could be changed due to the outcomes of interest. In this study, we excluded all subjects who had previously confirmed conditions which might change their dietary habits, such as dislipidemia, diabetes, hypertension, as well as CVD. Inverse causal relationship between dietary fiber and cardiovascular risk factors could thus be excluded in our study.

Another limitation is that the 24-h dietary recall method does not allow accurate assessments of infrequently consumed foods. In order to correct for such errors, nutrient intakes were corrected for within-person variability by applying the MSM method. Furthermore, we computed dietary fiber intake in the present study according to the China Food Composition Table, which so far don’t discriminate soluble from insoluble fiber. Therefore, the beneficial effect of dietary fiber on HDL-C is not clear, which may be elucidated in our future study.

Additionally, in the current study subjects were rural-to-urban workers from local factories rather than a population-based random sample. These subjects tended to have younger age, lower educational status and income than general population. However, no significant associations between dietary intake and education and economic status were observed. Therefore, these social economic differences between our subjects and the general population would be unlikely to significantly affect the DF–blood lipid association. Finally, it should be mentioned that the small sample size and differential recall by weight status may affect the result of this study.

## 4. Conclusions

Higher dietary fiber consumption is associated with increased plasma HDL-C in Chinese rural-to-urban male workers and with decreased TC/HDL-C ratio in Chinese rural-to-urban male and female workers. Our findings suggest dietary fiber may be beneficial for the prevention of cardiovascular diseases
